# Deep sub-wavelength nanofocusing of UV-visible light by hyperbolic metamaterials

**DOI:** 10.1038/srep38645

**Published:** 2016-12-07

**Authors:** Minkyung Kim, Sunae So, Kan Yao, Yongmin Liu, Junsuk Rho

**Affiliations:** 1 Department of Mechanical Engineering, Pohang University of Science and Technology (POSTECH), Pohang 37673, Republic of Korea; 2 Department of Electrical and Computer Engineering, Northeastern University, Boston, MA 02115, USA; 3 Department of Mechanical and Industrial Engineering, Northeastern University, Boston, MA 02115, USA; 4 Department of Chemical Engineering, Pohang University of Science and Technology (POSTECH), Pohang 37673, Republic of Korea

## Abstract

Confining light into a sub-wavelength area has been challenging due to the natural phenomenon of diffraction. In this paper, we report deep sub-wavelength focusing via dispersion engineering based on hyperbolic metamaterials. Hyperbolic metamaterials, which can be realized by alternating layers of metal and dielectric, are materials showing opposite signs of effective permittivity along the radial and the tangential direction. They can be designed to exhibit a nearly-flat open isofrequency curve originated from the large-negative permittivity in the radial direction and small-positive one in the tangential direction. Thanks to the ultraflat dispersion relation and curved geometry of the multilayer stack, hyperlens can magnify or demagnify an incident beam without diffraction depending on the incident direction. We numerically show that hyperlens-based nanofocusing device can compress a Gaussian beam down to tens-of-nanometers of spot size in the ultraviolet (UV) and visible frequency range. We also report four types of hyperlenses using different material combinations to span the entire range of visible frequencies. The nanofocusing device based on the hyperlens, unlike conventional lithography, works under ordinary light source without complex optics system, giving rise to practical applications including truly nanoscale lithography and deep sub-wavelength scale confinement.

Recently, nanophotonics has opened new realm of science and technology, providing breakthrough in many different fields including data processing, optical communications and holography. As one critical step in controlling light wave, nanofocusing of light is fundamental and essential in a broad range of applications such as nanolithography, surface-enhanced Raman spectroscopy (SERS), enhancement of nonlinear effects and single molecule detection. However, the diffraction limit in classical optics makes it very challenging to confine light into a deep sub-wavelength dimension. To overcome such fundamental limit, there have been active researches on nanoscale focusing, most of which have been realized by utilizing tapered plasmonic waveguides^1^,^2^,^3^,^4^,^5^, structural designs such as curved geometry^6^,^7^ and channel plasmon-polariton modes^8^. The tapered waveguide systems ensure strong field confinement and nanometer scale resolution, but they are limited by the spatial dispersion and fabrication difficulty. Meanwhile, nanofocusing based on structural design relies on geometrical shaping of the surface in micro or nanoscale and hence it is very challenging for implementation. In this paper, we present a different approach where photons are guided by extreme anisotropy of the medium, not by physical barrier as in tapered waveguide approaches. Our system is based on hyperbolic metamaterials to achieve nanofocusing which enables confining light into a deep sub-wavelength scale as well as super-resolution imaging with an ordinary light source at the UV and visible wavelengths, which are important range in nanolithography and imaging applications.

Metamaterials, artificial materials composed of the building blocks of deep sub-wavelength size and spacing, have been an intensive research subject. Metamaterials have shown various novel properties and applications such as negative refractive index^9^, cloaking^10^,^11^, sensing^12^, and imaging^13^,^14^,^15^ since Veselago first proposed the concept in 1968^16^. Hyperbolic metamaterials, one intriguing and special kind of metamaterials, are a highly anisotropic material with different signs of permittivity along different axes^17^,^18^. Originated from the extreme anisotropy, hyperbolic metamaterials have shown exotic properties with various applications, including all-angle negative refraction^19^,^20^,^21^, sub-diffraction-limited imaging^13^,^15^,^22^, enhancement of spontaneous emission^23^,^24^,^25^, photonic spin Hall effect^26^,^27^ and sub-wavelength waveguiding^28^,^29^. To realize hyperbolic metamaterials, we can use metallic nanowires embedded in a dielectric matrix or metal-dielectric multilayer. In this paper, we will focus on the latter case. As schematically illustrated in Fig. 1(a), the hyperbolic metamaterial consists of alternating layers of metal and dielectric with thickness much smaller than the wavelength, and hence the medium can be approximated as a homogeneous medium with effective optical parameters. When wave propagates parallel to the anisotropy axis, the effective permittivity satisfies the relation as follows,


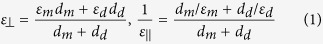


where *d* denotes thickness of each layer, the subscripts ⊥ and ∥ describe components perpendicular and parallel to the propagation directions, and the subscripts *m* and *d* indicate metal and dielectric, respectively. By adapting proper geometric parameters and materials, highly anisotropic permittivity can be achieved.

For transverse magnetic (TM) waves propagating in the multilayer metamaterials, the dispersion relation is given by


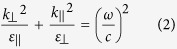


Here, the subscripts ⊥ and ‖ follow the same notation defined earlier. k is the wavevector, ε is the relative permittivity, ω is the angular frequency, and c is the speed of light in vacuum. At the frequency where the multilayer metamaterial has different signs of permittivity, the isofrequency surface exhibits hyperboloid shape as shown in Fig. 1(c) and (d), while naturally available anisotropic materials generally show ellipsoidal isofrequency surface (Fig. 1(b)).

## Results

Thanks to this extraordinary dispersion relation, hyperbolic metamaterials support TM waves with transverse wave vector *k*_‖_ larger than k_0_ (wave vector in vacuum). Such waves are evanescent waves that cannot propagate in air, but they can be converted into propagating waves through a mechanism of angular momentum conservation in hyperlens^30^,^31^. Using these properties, super-resolution imaging has been achieved by using hyperlens, a cylindrical^13^,^15^,^22^ or a spherical^32^ multilayer hyperbolic metamaterials from many groups.

**Figure 2:**
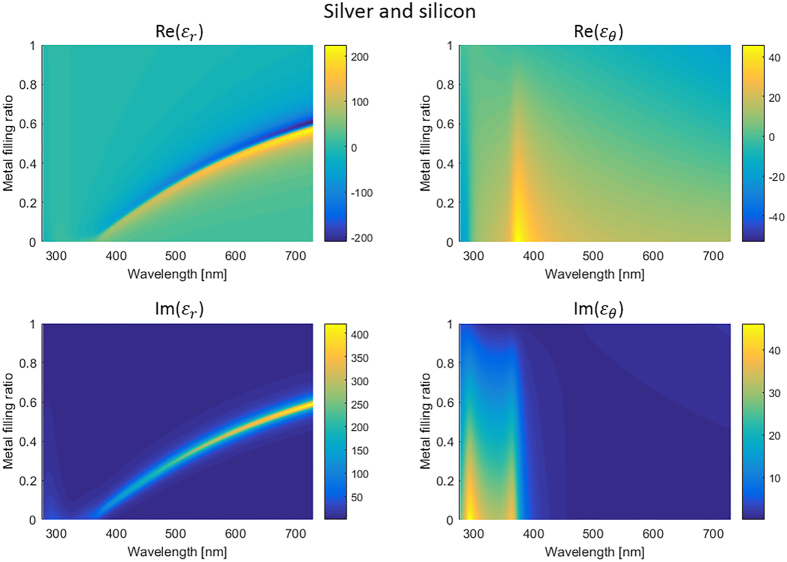
Effective permittivity of multilayer composed of silver and silicon.

Since the effective permittivity depends on the optical properties of the metal and dielectrics components and their filling ratio as described in equation (1), different material compositions yield different working frequency ranges. One example is shown in Fig. 2 for silver and silicon. Metal and dielectrics are properly determined to provide hyperbolic dispersion as flat as possible and high transmission for nanofocusing and super-resolution applications. For flat dispersion, real part of effective permittivity should be highly negative in radial direction and slightly positive in tangential direction, as implied in equation (2). The effective permittivity should also have small imaginary part in both radial and tangential directions in order to allow high transmission. Although realizing hyperbolic dispersion using semiconductors instead of novel metals has been reported in infrared wavelength range^19^,^33^, material combinations conventionally used in visible frequency are limited to noble metals such as silver and gold and high index dielectrics because noble metals exhibit low losses whereas other metals such as aluminum are not used due to the relatively high losses.

**Figure 3:**
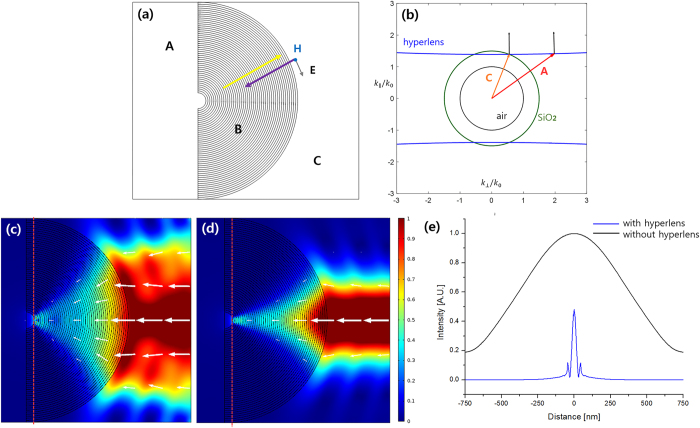
Schematic and 2D simulation of the hyperlens-based nanofocusing. (**a**) Schematic design. Media denoted by A, B and C are air, hyperlens and silicon dioxide, respectively. Yellow and purple arrows denote Poynting vector of outgoing and incoming TM waves, respectively. (**b**) Two-dimensional isofrequency curve for TM waves in hyperlens consisting of silver and silicon (blue), compared to the isofrequency curve of silicon dioxide (green) and air (black) at wavelength 560 nm. Red and orange arrows marked by A and C indicate wave vectors supported by the media of A and C in (**a**), and the black arrows indicate the direction of group velocities in the hyperlens. (**c**) and (**d**) Intensity profile in the hyperlens composed of silver and silicon with incident beam radius of (**c**) 900 nm and (**d**) 400 nm and white arrows denote Poynting vector. (**e**) Normalized intensity along red dotted line when the incident beam has radius of 400 nm.

Figure 3(a) shows a schematic design where media marked by A, B and C are air, hyperlens and silicon dioxide, respectively. The hyperlens is composed of alternatively stacked metal and dielectric in which the thickness of the each layer is set to 15 nm and outer and inner radius is 750 nm and 60 nm, respectively. If the hyperlens has ultraflat iso-frequency curve for TM waves as shown in Fig. 3(b), it can support broad range of transverse component of the wave vector and compress or expand transverse wave vector without diffraction as beam passes the hyperlens depending on its propagation direction. As waves propagate along the radial direction from inner medium (A) to outer medium (C) (yellow arrow in Fig. 3(a)), the transverse wave vector decreases from the red arrow to the orange arrow in Fig. 3(b). Therefore, evanescent waves become propagating without diffraction or distortion of the original beam shape since the direction of the group velocities which are represented by the black arrows in Fig. 3(b) barely change. Moreover, because the group velocities in the hyperlens have nearly zero transverse components, the waves propagate along the radial direction, resulting magnification of the beam size. In other words, the role of the hyperlens is to convert evanescent waves into propagating waves and to magnify the image. However, if light passes the hyperlens inversely (purple arrow in Fig. 3(a)), radial propation direction leads to compression of the incoming waves, and the incident beam will be focused into an area below the diffraction limit^34^,^35^. Moreover, since the propagation of light depends on radial group velocity, highly anisotropic elliptical dispersion also supports nanofocusing with super-resolution.

Figure 3(c) and (d) are 2D simulation results of hyperlens-based nanofocusing using Finite Element Analysis since it has rotational symmetry. The incident beam is a TM-polarized Gaussian beam with beam radius of 900 nm in (c) and 400 nm in (d) both of which are easily achievable using conventional optical systems. While the incident beam exhitibits a full width at half maximum (FWHM) of 817 nm in free space, the beam in the hyperlens is compressed as it propagates, resulting in 34 nm FWHM which corresponds to 0.04 of that without hyperlens. Although the intensity, which corresponds to efficiency, is reduced to half, it is still relatively high compared to imaging application due to the localization of field.

**Figure 4:**
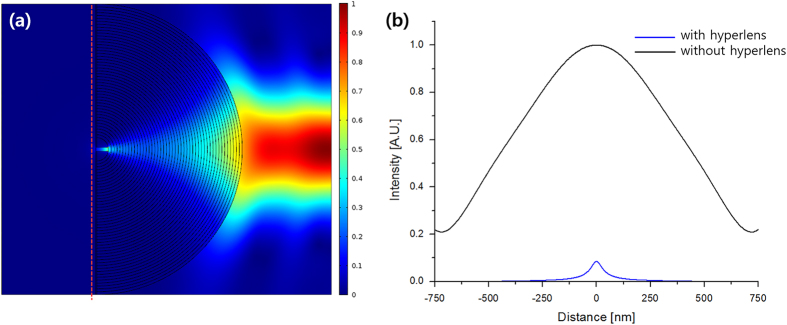
Hyperlens-based nanofocusing where chrome layer with slit is added to the inner surface. (**a**) Intensity profile in the hyperlens consisting of silver and silicon at wavelength of 560 nm. (**b**) Normalized intensity along the vertical line spaced 20 nm from the chrome layer which is indicated as red dotted line. The width of the slit is set to 20 nm.

This sub-wavelength focusing can be used in applications such as nanopatterning by adding a layer with slit to the inner surface (Fig. 4(a)). Light has been an attractive source of lithography for its easy accessibility and low cost, but diffraction is a natural and fundamental phenomenon obstructing nanoscale lithography. Utilizing hyperlens, which can confine light in sub-wavelength area, a new concept of nanoscale lithography under simple UV and visible light illumination beyond the current photolithography is achievable. Intensity along the line spaced 20 nm from the layer which is marked as red dotted line is shown in Fig. 4(b). Although intensity decreases rapidly as it goes away from the focal point since it is evanescent, it is still enough for lithography application. Therefore, hyperlens is a very promising candidate for deep sub-wavelength patterning of few nanometers scale without complicated optics systems, and it can be also integrated with more complex lithography system such as flying head lithography utilizing highly focused near-field focused light^36^.

**Figure 5:**
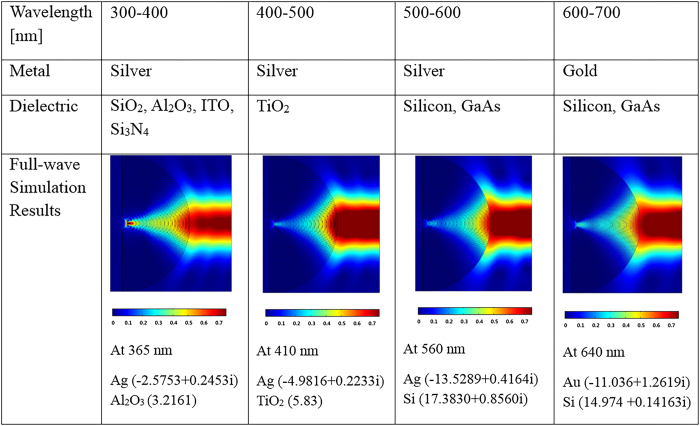
Materials according to the operating wavelength spanning 300 nm to 700 nm where metal filling ratio is 1/2. Color maps indicate the magnitude of Poynting vector. Wavelength and materials (permittivity) used for the simulation are shown.

Furthermore, this diffraction-unlimited focusing can be achieved in broad range of visible frequency by changing the materials. Figure 5 shows possible material combinations in the UV and visible range and its full-wave simulation where color map shows magnitude of Poynting vector. Here, metal filling ratio is fixed to 1/2 in all simulations. The wavelength, materials and their permittivities used in each simulation are specified under the simulation result. (The material properties are taken from literature^37^ for gold and experimentally measured value for the remaining.) Working frequency range can be, however, further expanded by diversifying the material combinations which has not been used widely such as conventional semiconductors. Also, hyperlens can have additional degrees of freedom by adapting graphene or phase change materials (PCM). High optical tunability of graphene-based hyperlens has been recently demonstrated in the mid-infrared region by controlling the applied voltage^38^,^39^, and PCM also proved characteristic optical properties which is tunable in the vicinity of the phase transition by tuning temperature in near-infrared regime^40^,^41^. If one can apply these schemes in the visible range, single material combination can cover whole range of optical frequencies by simply tuning voltage or temperature, opening new possibilities in both nanofocusing and imaging area.

In conclusion, hyperlens–based nanofocusing system provides both deep sub-wavelength confinement of light and super-resolution imaging by controlling the spot size in nanometer scale without complex optics system such as electron beam lithography, ion beam lithography and deep-UV photolithography. We have numerically showed light localization originated from nearly-flat hyperbolic dispersion and suggested various material combinations to cover parts of UV and whole visible range. Introducing tunable materials such as graphene and PCM will bring useful applications which remains as future works. Although fabrication of the hyperlens system is challenging because of their requirement of geometry definition and multi-level thin-film deposition process, it could be further improved with the on-going researches in scalable nanofabrication processes such as roll-to-roll system and pattern transfer techniques. Further, the proposed deep sub-wavelength nanofocusing property of hyperlens can be applied to not only low-cost and high-resolution nanoscale lithography, but also several other interesting applications such as SERS and single molecule level imaging with the extremely focused near-field light.

## Methods

We used COMSOL Multiphysics 5.0 for numerical simulation.

## Additional Information

**How to cite this article**: Kim, M. *et al*. Deep sub-wavelength nanofocusing of UV-visible light by hyperbolic metamaterials. *Sci. Rep.* **6**, 38645; doi: 10.1038/srep38645 (2016).

**Publisher's note:** Springer Nature remains neutral with regard to jurisdictional claims in published maps and institutional affiliations.

## Figures and Tables

**Figure 1:**
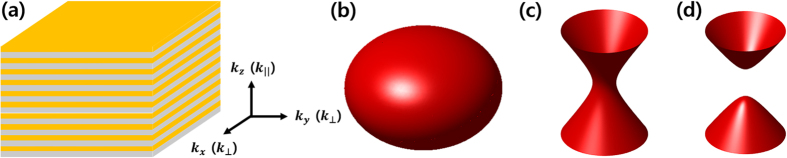
(**a**) Schematic of metal-dielectric multilayer stack. Isofrequency surface of TM waves with (**b**) both *ε*_⊥_ and *ε*_‖_ positive but not identical, (**c**) *ε*_⊥_ negative and *ε*_‖_ positive, and (**d**) *ε*_⊥_ positive and *ε*_‖_ negative. The surface normal of multilayer is defined as z-direction.
